# On the role of polymeric hydrogels in the thermal response of gold nanorods under NIR laser irradiation†

**DOI:** 10.1039/d3na00353a

**Published:** 2023-08-28

**Authors:** Elisa Lacroce, Leonardo Bianchi, Laura Polito, Sanzhar Korganbayev, Alessandro Molinelli, Alessandro Sacchetti, Paola Saccomandi, Filippo Rossi

**Affiliations:** a Department of Chemistry, Materials and Chemical Engineering “Giulio Natta”, Politecnico di Milano via Mancinelli 7 20131 Milan Italy filippo.rossi@polimi.it +39-02-2399-3145; b Department of Mechanical Engineering, Politecnico di Milano via Giuseppe La Masa 1 20156 Milan Italy paola.saccomandi@polimi.it +39-02-2399-8470; c Consiglio Nazionale delle Ricerche, CNR-SCITEC via Gaudenzio Fantoli 16/15 20138 Milan Italy

## Abstract

Hydrogels are 3D cross-linked networks of polymeric chains designed to be used in the human body. Nowadays they find widespread applications in the biomedical field and are particularly attractive as drug delivery vectors. However, despite many good results, their release performance is sometimes very quick and uncontrolled, being forced by the high *in vivo* clearance of body fluids. In this direction, the development of novel responsive nanomaterials promises to overcome the drawbacks of common hydrogels, inducing responsive properties in three-dimensional polymeric devices. In this study, we synthesized and then loaded gold nanorods (Au NRs) within an agarose-carbomer (AC)-based hydrogel obtained from a microwave-assisted polycondensation reaction between carbomer 974P and agarose. The photothermal effect of the composite device was quantified in terms of maximum temperature and spatial–temporal temperature distribution, also during consecutive laser irradiations. This work shows that composite Au NRs loaded within AC hydrogels can serve as a stable photothermal treatment agent with enhanced photothermal efficiency and good thermal stability after consecutive laser irradiations. These results confirm that the composite system produced can exhibit an enhanced thermal effect under NIR laser irradiation, which is expected to lead to great therapeutic advantages for the localized treatment of different diseases.

## Abstract 2

Hydrogels are 3D cross-linked networks of polymeric chains designed to be used in the human body.

## Graphical abstract

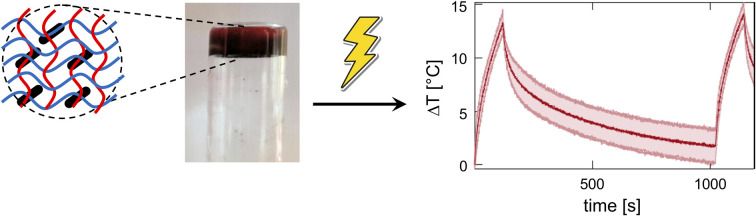

## 1. Introduction

The light source is one of the most frequent external stimuli used in medicine and has been reported in many drug delivery systems integrated into technologies like lasers, lamps, and light-emitting diodes (LEDs)^1–3^ Among them, near-infrared (NIR) mediated treatments showed great promise as a therapeutic strategy in different fields.^4–6^ They present several advantages like minimal invasiveness, short treatment time, and rapid recovery.^7,8^ Moreover, another great advantage includes their higher safety with respect to UV light and that they can be used as remote stimuli, are non-invasive, and are almost easy to use. In this direction, several studies underlined the importance of semiconductor-based, carbon-based, and plasmonic metal-based nanoparticles that can convert excitation energy into heat.^9–11^ Indeed, colloidal systems currently play a pivotal role in precise targeting with consequent thermal effects minimizing side effects to surrounding tissues.^12,13^ Among them, gold-based colloids showed great promise due to their easiness in terms of preparation, high biocompatibility and responsive ability to NIR exposure.^14–16^ One of the key characteristics of gold nanoparticles (Au NPs) is the surface plasmonic resonance (SPR) which can be used to transform an electromagnetic surface into heat. Indeed, using electromagnetic radiation on NPs it is possible to excite the surface electrons, which thermalize with the phonons releasing heat.^8,17,18^ Also, the size and the shape of Au colloids are fundamental and can cause the shift of the absorption coefficient from the visible to the NIR spectral range if nanorods are used instead of spherical shaped NPs. The light used, in the NIR region, presents several advantages like a better penetration depth inside biological tissue and can be used to have an effect under the skin barrier. Then, the heat generated can be used in different medical treatments like cancer ablation or drug delivery. In the latter, the heat generated by the interaction between light and Au colloids can lead to morphological modification of the NPs with consequent drug release. Despite the very good results obtained with these nano-objects, several challenges remain like the quick escape of nanoparticles from the target site if they are not properly confined.^19–21^

This can limit the therapeutic effects on one side and cause accumulation problems in healthy tissues on the other one. These challenges can be solved by confining Au colloids within a 3D polymeric network. In this direction, in the last few years, hydrogels have attracted a lot of interest in the field of biomedical research.^22–25^ They are polymeric 3D structures able to swell and contain large amounts of water mimicking very well the extracellular matrix. Hydrogel networks can therefore entrap Au NPs avoiding their uncontrolled release within the body and, if properly designed, become NIR-sensitive. The main issue is that polymers are generally not sensitive to NIR light and the loading of gold colloids within them can decrease their performances as photothermal agents.^26,27^ Hence, the aim of this study is to examine the NIR photothermal activity of gold nanorods (Au NRs) embedded within an agarose-carbomer (AC)-based hydrogel developed by our laboratory.^28,29^ The synthesis of hydrogel consisted of microwave-assisted polycondensation reactions between carbomer 974P (branched polyacrylic acid) and agarose, a natural polysaccharide. They are both biocompatible and FDA-approved polymers and they can crosslink in aqueous media showing great promise in the biomedical field. Gold NRs were synthesized and fully characterized in terms of size, morphology, composition, and thermal stability, as well as their efficient entrapment avoiding their release from the matrix. The photothermal effect was quantified in terms of maximum temperature and spatial–temporal temperature distribution, also during consecutive laser irradiations. This work shows that composite Au NRs/AC-HG can serve as a stable photothermal treatment agent with high and enhanced efficiency, which is of great potential for further clinical translations to combat difficult-to-treat diseases.

## 2. Materials and methods

### 2.1. Synthesis of Au NRs

The synthesis was performed following the synthesis of B. Wu (7). Briefly, a seed and a growth solution were created. For the growth solution, 0.72 g of CTAB and 0.098 g of sodium oleate were dissolved in 20 mL of milli Q and maintained under stirring at 90 °C for 90 minutes. Then, the mixture was cooled down to room temperature. 20 mL of milli Q water and 2 mL of HAuCl_4_ solution 10 mM were added to the solution which remained under stirring for 90 minutes at 30 °C. The colour of the solution changed from orange to transparent. For the seed solution, 1 mL of CTAB 0.1 M was mixed with 43 μL of HAuCl_4_ 10 mM. The solution becomes a yellow/orange colour. Then, 60 μL of an ice-cooled solution, prepared by dissolving 1.89 mg NaBH_4_ in 0.5 mL milli Q water, was added and the solution was maintained under stirring for 30 minutes at 30 °C. A solution of HCl (37 wt%) was added to the growth solution and after 15 minutes 64 μL of ascorbic acid solution 0.1 M and 400 μL of AgNO_3_ 10 mM were added. Immediately, 32 μL of seed solution was added and after 30 seconds the stirring was stopped, and the solution was maintained for 12 hours at 30 °C. The purification was performed by removing the supernatant after centrifugation at 6000 rpm for 15 minutes. A second centrifugation was performed with the remaining CITAB solution and, in the end, the recovered NRs were diluted with milli Q water.

### 2.2. Synthesis of NP-loaded hydrogels

Polymeric solution was achieved by mixing polymer powders of agarose and carbomer 974P in phosphate buffered saline (PBS) solution under batch conditions, adding a mixture of cross-linking agents made of propylene glycol and glycerol along with 1 N NaOH (reaction pH was kept neutral).^28,30,31^ The gelation onset was achieved by means of electromagnetic stimulation (500 W irradiated power) heating in a ratio of 1 min per 10 mL of polymeric solution at 80 °C. Microwave-enhanced chemistry is based on the efficient heating of materials by “microwave dielectric heating” effects.

During the hydrogel (HG) cooling phase, slightly above 37 °C, HGs in the sol state were mixed with Au NRs (3 mL of NP dispersion per mL of hydrogel): this procedure allows solute loading still during the sol state, *i.e.*, before sol/gel transition. The loaded HG-NR systems were cast in standard plastic 48-well cell culture plates, 0.5 mL per well (diameter = 1.1 cm). Gelation studies were performed using an inverter tube test compared with blank HGs.

#### 2.2.1. Transmission electron microscopy (TEM)

The size and morphology of produced NRs were confirmed by TEM (using an EFTEM Leo 912AB, at 80 kV, by Karl Zeiss, Jena, Germany). Samples were prepared by placing a 5 mL drop of NP dispersion on a Formvar/carbon-coated copper grid and dried overnight. Digital images were acquired by using a charge-coupled device (CCD; Esi Vision Proscan camera).

#### 2.2.2. Dynamic light scattering (DLS)

The hydrodynamic diameters of the NRs under investigation were measured by using a Malvern Zetasizer Nano ZS90 DLS. For each sample, a scattering angle of 173°, a temperature of 25 °C and an equilibration time of 30 s were used.

#### 2.2.3. UV-analysis

The synthesized Au NRs were characterized by using UV-vis absorption spectra recorded using a UV-vis spectrophotometer (Shimadzu UV-1800, Kyoto, Japan) over the wavelength range from 300 to 1000 nm. The samples were measured at room temperature in a 1 cm-optical path plastic cuvette, in which a gel of 3 mm thickness was fixed with water.

### 2.3. Hydrogel characterization

#### 2.3.1. Fourier-transform infrared spectroscopy (FT-IR)

Hydrogel samples, after being left to soak for 24 hours in excess of solvent, were freeze-dried and laminated with KBr.

FT-IR transmission spectra were recorded using a Thermo Nexus 6700 spectrometer coupled to a Thermo Nicolet Continuum microscope equipped with a 15× Reflachromat Cassegrain objective at a resolution of 4 cm^−1^ using the KBr pellet technique.

#### 2.3.2. Scanning electron microscopy (SEM)

Scanning electron microscopy analysis was performed on gold sputtered samples at 10 kV with Evo 50 EP Instrumentation (Zeiss, Jena, Germany). To preserve the actual morphology of the hydrogel under complete swelling, freeze-drying (for 24 hours) was applied to remove all the liquid phase by sublimation. Because of the low operating values of temperature and pressure, the polymer chains were expected to retain the same conformation they had in wet conditions. Comparative evaluation of the superficial and internal morphology of investigated samples was carried out.

#### 2.3.3. Thermogravimetric analysis (TGA)

Experiments were performed using an SDT Q6000 PerkinElmer. TGA sample weights in these studies ranged from 2 to 10 mg. Under a nitrogen purge, a heating rate of 10 °C min^−1^ was used to scan from 30 to 900 °C. The temperature of 900 °C was maintained for the last 30 minutes.

#### 2.3.4. Differential scanning calorimetry (DSC)

DSC experiments were performed with a PerkinElmer DSC 7 under nitrogen purge gas. All samples were subjected to heating and cooling cycles consisting of a ramp from −60 °C to 250 °C at 10 °C min^−1^.

#### 2.3.5. Gelation time

Gelation was assessed using the inverted tube test. Two mL microcentrifuge tubes (Fisher Scientific, Ottawa, ON, CA) were filled with 900 μL PBS and equilibrated to 37 °C. One hundred μL of the polymer solution was injected into the bottom of the tube and incubated at 37 °C. At different intervals, tubes were inverted to observe if the gel flowed. The time at which the gel did not flow was recorded as the gelation time.

### 2.4. NR release from hydrogelS

Three samples were put in excess of PBS and aliquots were collected at defined time points, while the sample volume was replaced by fresh PBS, in order to avoid mass-transfer equilibrium between the gel and the surrounding solution. The percentage of NRs released was measured through DLS analysis.^30^

### 2.5. Measurement of photothermal performance of Au NR-loaded hydrogel samples

The thermal effect of the Au NR-loaded hydrogel samples was evaluated by measuring the temperature of the samples under NIR irradiation. The latter was obtained with a diode laser source emitting light at a wavelength of 808 nm in a continuous wave mode.^32^ All the samples were placed in 1.5 mL Eppendorf tubes, and two concentrations of Au NRs, *i.e.*, 0.00182 and 0.00091 mg mL^−1^, were selected. For control measurements, samples of hydrogel with distilled water were used (the hydrogel was diluted with distilled water to obtain the same density of the Au NR samples), in order to infer the effective contribution of Au NRs in the sample heating. The laser light was guided to the samples through a 300 μm-diameter quartz multimode optical fiber (GSI Lumonics), the focal spot of the laser beam was set at the center of the suspension to ensure uniform distribution. The effect of the output laser power on the thermal response of the samples was taken into account; thus, the experiments were performed at 3 W, 4 W and 5 W. Each constant laser power value was maintained for 120 s in all the tests. For each laser setting, the measurement was repeated three times, each time on a new sample. The photothermal stability of Au NR-loaded hydrogel samples was evaluated by monitoring the temperature change during three consecutive laser on-and-off cycles performed at 5 W for 120 s with Au NR concentration of 0.00182 mg mL^−1^, with a cooling time of 100 s between successive cycles. Also for this analysis, three different samples were involved, and, on each, three consecutive laser on-and-off cycles were performed. Temperature measurements were performed during the laser irradiation procedure using a fiber Bragg grating (FBG) array – optical fiber having a chain of FBGs along its length, where each FBG acts as a temperature sensor.

The FBG array (FiSens GmbH, Braunschweig, Germany) consists of 10 FBGs: each sensor has an active length of 1 mm, and the edge-to-edge distance between adjacent FBGs is equal to 1 mm. As a result, the FBG array has a 2.0 mm spatial resolution along a 19 mm sensing length, which is suitable to cover the whole length of the 1.5 mL Eppendorf tubes which were used for the experiments. The optical signal of the FBG array (10 reflected spectral peaks, *i.e.*, Bragg wavelengths [nm]) was monitored using a HYPERION si255 interrogation unit (Micron Optics, Atlanta, USA, precision of 1 pm, corresponding to 0.1 °C). The temperature reconstruction is based on the fact that temperature changes (Δ*T*) of FBGs are proportional to shifts of corresponding Bragg wavelengths. Where the coefficient of proportionality, the thermal sensitivity (equal to ∼11.4 pm °C^−1^), was obtained through static calibration.^33^ A custom-made LabVIEW software for real-time measurements was used, and the post-processing analysis was made with MATLAB. The start of measurements of the optical signal was 10 s before each NIR irradiation. This logic has been implemented to make sure that we acquire the whole heating phase of each sample. After the laser was turned off, the acquisition of the optical signal was maintained for 30 s more (cooling phase).

### 2.6. Statistical analysis

Experimental data were analyzed using a one-way ANOVA test followed by Bonferroni's *post hoc* test. Statistical significance was set to *p* value < 0.05. The results are presented as mean value ± standard deviation. The results of Δ*T* measurements are reported as mean ± standard deviation, calculated on the three different samples involved in the experimental analyses.

## 3. Results and discussion

### 3.1. Preparation and characterization of Au NRs

**Fig. 1:**
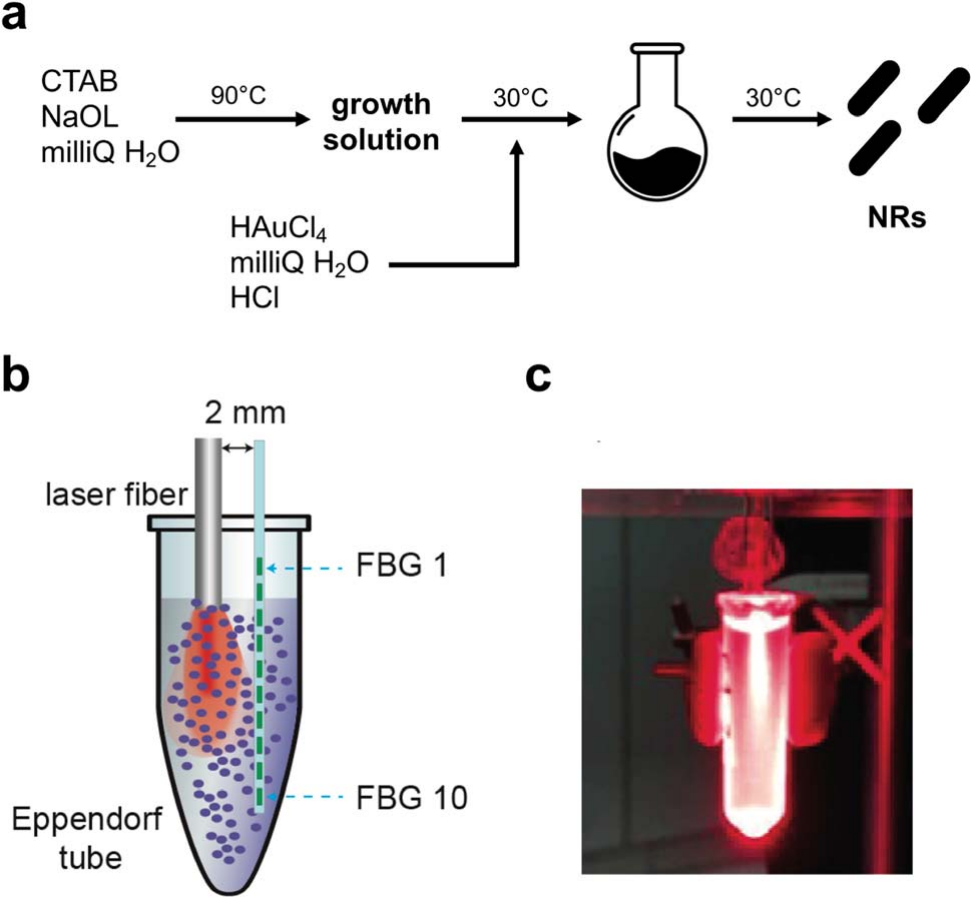
(a) Schematic illustration of the synthetic route used to produce Au NRs; (b) experimental setup used to perform NIR irradiation of the Au NR samples and to measure the attained temperature distribution in real time; (c) picture of the sample under NIR irradiation. The visible red light is emitted from a pilot source.

The synthetic steps used to prepare Au NRs are presented in Fig. 1a. Synthesis of fine nano-sized rods was carried out starting from a growth solution then added with chloroauric acid as presented in detail in the Materials and methods section. The experimental setup used to assess the photothermal effect of the samples is depicted in Fig. 1b. The Au NR-loaded hydrogel samples were placed in Eppendorf tubes, useful to adjust the laser fiber and the 10 FBG temperature sensors inside the samples. Fig. 1c shows the picture of the sample undergoing NIR irradiation, with the support of a visible pilot light.

**Fig. 2:**
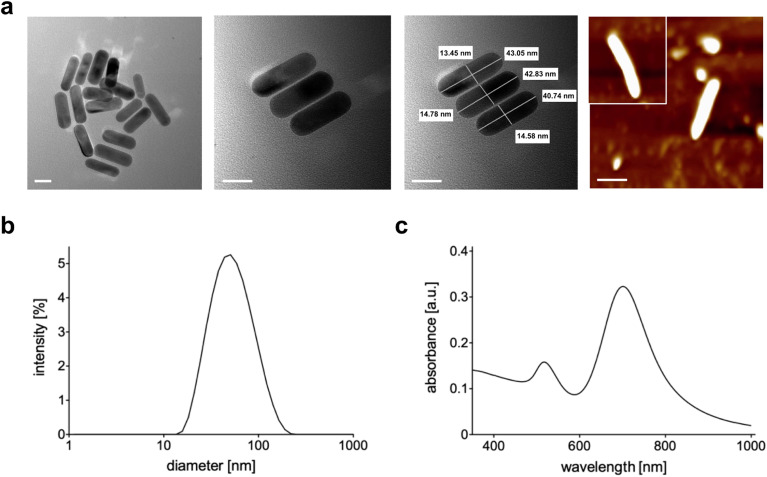
(a) TEM and AFM (last panel) images of Au NRs (scale bar = 20 nm); (b) DLS analysis of Au NRs; (c) absorption spectrum of Au NRs.

The produced NRs were then characterized in terms of size, shape and stability. Transmission electron microscopy (Fig. 2a) confirmed the formation of rods with dimensions of 45 nm × 15 nm approximately. The final shape obtained was also confirmed by AFM (Fig. 2a last panel) while the dimension was by dynamic light scattering (DLS, Fig. 2c). As is well-known, DLS analysis considers every colloid as round shaped and so the value obtained from the graph is about the biggest dimension (approximately 50 nm). All three techniques used showed the absence of aggregation and so the successful formation of stable and well-separated Au NRs. Fig. 2c shows the measured absorption spectrum of Au NRs with the two typical adsorption peaks around 490 nm and 700 nm attributed to the typical TSPR and LSPR of Au NRs of similar dimensions.^7,34^

**Fig. 3:**
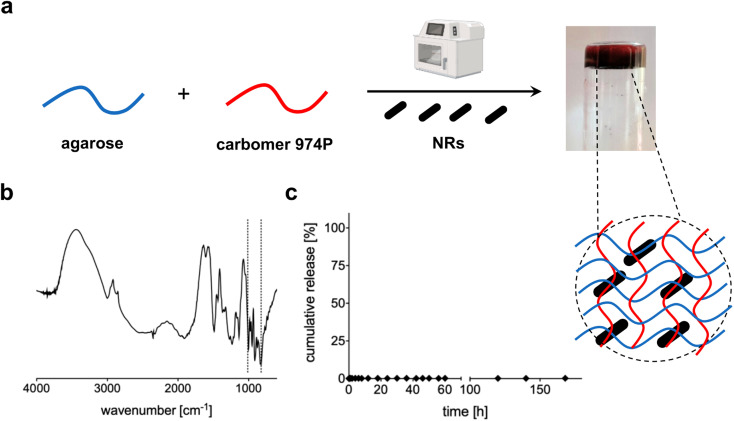
(a) Schematic representation of the preparation of AC hydrogels loaded with Au NRs; (b) FT-IR spectrum of AC hydrogels loaded with Au NRs; (c) cumulative release of Au NRs from the 3D polymeric network of AC hydrogels.

Au NRs were then loaded within the 3D network of agarose-carbomer-based hydrogels as visible in Fig. 3a. Agarose and carbomer form a chemical cross-linked hydrogel after microwave-assisted polycondensation between hydroxyl groups of agarose and carboxyl ones of carbomer. They form ester groups responsible for the formation of the polymeric network. This synthesis is based on the high efficiency of the heating that takes place using the effect called “microwave dielectric” where microwave radiation is converted into heat. Key advantages behind the use of microwave radiation are the absence of catalyst, initiator and organic solvents. Before complete gelation, when the polymer solution is yet at sol state, Au NRs were added and then gelation took place. Gelation time was measured with or without the presence of NRs, showing that their presence does not influence the time needed to form a gel that is around 7 minutes (ESI†). The absence of influence of NRs in physical properties of the final hybrid device is also evident from TGA and DSC analyses (ESI†); FT-IR analysis (Fig. 3b) shows the characteristic peaks of Au (850–1000 cm^−1^)^35,36^ whereas the wavenumber range 3600–2900 cm^−1^ corresponds to the stretching vibration of –OH groups with partial overlap with the peak of C–H stretching at 2950–2850 cm^−1^. The peaks at 1700 cm^−1^ and 1560 cm^−1^ correspond to carboxylates groups, respectively symmetric and asymmetric stretches. In Fig. 3c, the release profile of Au NRs from the hydrogel network is presented, evidencing that nanorods are entrapped within the matrix and cannot escape until the complete degradation that *in vivo* takes place after months.^37^

**Fig. 4:**
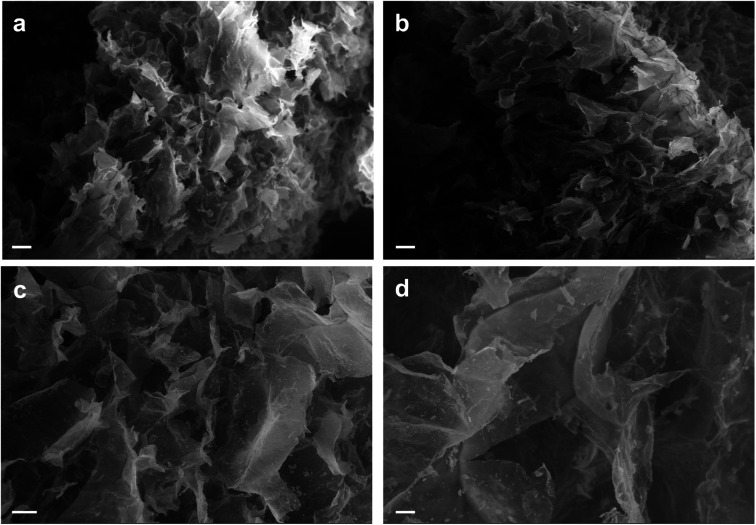
SEM analysis of AC hydrogels loaded with Au NRs (a and c) and neat AC hydrogels (b and d). Scale bars: 200 μm (a and b); 100 μm (c and d).

To observe the 3D structure of the hydrogels produced we used SEM to compare AC hydrogels loaded with Au NRs (Fig. 4a and c) together with neat AC hydrogels (Fig. 4b and d). The samples were analyzed after being prepared using a freeze-drying method that can maintain the same structure present in wet state even under dry conditions. Indeed, the removal of water can disrupt the structural stability with consequent collapse of the structure. Our results underlined that these hydrogels present a highly entangled and interconnected structure with big pores that contain smaller ones in accordance with previous studies.^38,39^ In addition, from the SEM analysis, we can underline that the presence of Au NRs cannot alter the polymeric network (other images in ESI†).

### 3.2. Photothermal response of Au NR-loaded hydrogel samples

**Fig. 5:**
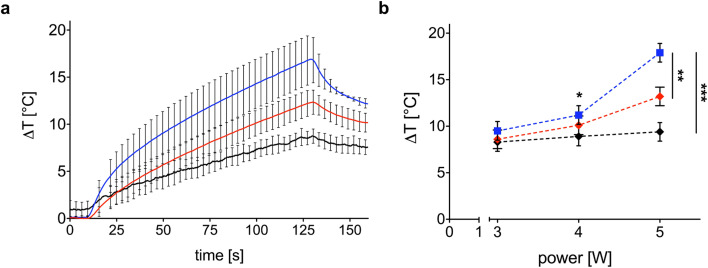
(a) Trend of maximum Δ*T* measured to evaluate the photothermal effect of the Au NR-loaded hydrogel samples (0.00182 mg mL^−1^, blue line; 0.00091 mg mL^−1^, red line) *vs.* Δ*T* measured in the control samples (black line), both irradiated at 5 W. The bars represent the standard deviation calculated on the three repetitions; (b) effect of the laser power on the measured Δ*T*. The Δ*T* achieved with the irradiation at 5 W shows a statistical difference with respect to the Δ*T* obtained at 3 W (*) *p* < 0.05. (**) *p* < 0.01. (***) *p* < 0.001.

A key point is represented by the photothermal response of AC hydrogels loaded with Au NRs. Their photothermal performance is quantified in terms of the maximum temperature induced by the laser irradiation at 808 nm and measured by the FBG sensor placed closer to the laser fiber tip (Fig. 5). Among the different temperature monitoring techniques, FBG sensors were adopted as reliable sensing solutions for the specific application. Indeed, the miniaturized dimension, the immunity from electromagnetic interferences, and the possibility of performing quasi-distributed temperature measurements, thanks to multiple sensing points within a single fiber and multiplexing capability, represent captivating features of FBGs.^40–42^ Furthermore, the good metrological characteristics of the employed fiber optic sensors, *i.e.*, 2.0 mm spatial resolution and 0.1 °C accuracy, allow for the accurate detection of temperature gradients in the specimens.

Besides, FGB sensor-based thermometry overcomes several drawbacks related to the application of traditional and often employed temperature measurement techniques, such as measurement systems based on thermocouples and thermographic imaging, for the evaluation of the photothermal performance of Au NR-loaded hydrogel samples undergoing laser irradiation. Thermocouples allow for single-point measurements; hence, it is necessary to use more than one if multiple measurements, such as those obtained with FBG sensors, are desired. Additionally, due to the laser light absorption by the metallic components of the thermocouples, measurements conducted using thermocouples for evaluating laser-induced photothermal effects may be prone to errors, particularly overestimation of the sample temperature.^43^ On the other hand, thermographic imaging, despite the advantage of non-invasive measurement, allows for the measurement of the surface temperature of the specimen. Therefore, in our application, the employment of thermographic imaging could lead to a measurement of the temperature of the Eppendorf tube in which the hydrogel is located, deviating from the actual temperature of the Au NR-loaded hydrogel sample. For these reasons, our system for measuring temperature change in samples was based on fiber optic FBG sensors. Despite the unique advantages they present, the application of these sensors for evaluating the laser-induced photothermal effect in hydrogels containing nanoparticles is still novel and limited in the literature of the field.^32,44^

The photothermal response of the samples is dependent upon Au NR concentration and laser power. Fig. 5a shows the temporal trend of maximum Δ*T* obtained with the Au NR-loaded hydrogel samples at two different Au NR concentrations (0.00182 mg mL^−1^, blue line; 0.00091 mg mL^−1^, red line). Indeed, 0.00182 mg mL^−1^ of Au NRs triggers a mean Δ*T* of 16.9 °C after 120 s of irradiation (black line in Fig. 5a), while, decreasing the concentration of Au NRs loaded within the hydrogel network to 0.00091 mg mL^−1^ (red line in Fig. 5a), the mean Δ*T* measured is 13.2 °C. In comparison with the mean Δ*T* achieved by the control sample (8.6 °C), the higher Au NR concentration provides a temperature increase of 96%, *vs.* 44% obtained with the lower Au NR concentration in the hydrogel matrix.

Laser power is one of the key parameters in photothermal therapy,^45,46^ and its effect on Au NR-loaded hydrogel samples at both concentrations is depicted in Fig. 5b. While in the control samples, the power is slightly affecting the maximum Δ*T* and with a linear trend (Fig. 5b, black curve), increasing the laser power from 3 to 5 W in the hydrogel samples loaded with Au NRs leads to a non-linear Δ*T* increase (Fig. 5b, blue and red curves), which is in accordance with the temperature variation trends observed in previous investigations on the heating performances of Au NRs in water solutions.^45^

**Fig. 6:**
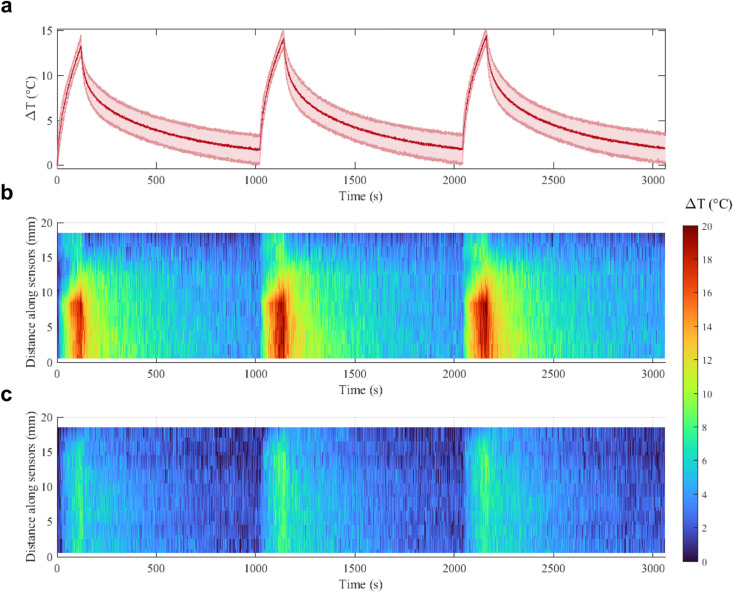
On-off cycles: (a) maximum Δ*T* measured in the Au NR-loaded hydrogel samples undergoing 808 nm irradiation at 5 W. The results are shown as mean value and standard deviation bands, calculated on the three samples. Temperature distribution in the (b) Au NR-loaded hydrogel samples and (c) control samples during the first, second, and third cycles.

With a concentration of 0.00182 mg mL^−1^ of Au NRs and after 120 s irradiation (Fig. 5b, blue curve), the Δ*T* achieved at 5 W is almost two times higher than the Δ*T* at 3 W (16.9 °C *vs.* 8.8 °C, temperature increase of 92%). At 4 W, Au NRs allow achieving 10.6 °C *vs.* 8 °C measured in the control samples (temperature increase of 32%). On the contrary, irradiation with 3 W provides similar thermal effects in both Au NR-loaded hydrogel and control samples, with a Δ*T* of 8.8 °C *vs.* 7.4 °C, respectively (temperature increase of 19%). Similar behavior can be observed for the hydrogel networks embedding 0.00091 mg mL^−1^ of Au NRs (Fig. 5b, red curve). Moreover, the Δ*T* achieved with the laser irradiation at 5 W and with higher Au NR concentration shows a statistical difference with respect to the Δ*T* obtained for the same power in both the control and hydrogel-based samples embedded with Au NRs at lower concentrations. Also, for 0.00182 mg mL^−1^ of Au NRs, the Δ*T* achieved with the irradiation at 5 W shows a statistical difference compared with the Δ*T* obtained at 3 W. The laser on-off-cycles tests allowed to evaluate the potential modification of the Au NR-loaded hydrogel samples (concentration of Au NRs equal to 0.00182 mg mL^−1^) undergoing consecutive NIR irradiation cycles. The results in Fig. 6, presented in terms of both peak temperatures (Fig. 6a) and temperature distribution (Fig. 6b), show that the trend and magnitude of temperature change have no observable alteration after three irradiation cycles. It is worth noting that the laser irradiation of the sample leads to a temperature gradient, thus, to a non-uniform temperature distribution inside the hydrogel (Fig. 6b).

For this reason, sensors at position 8–9 mm along the sensor array, which are the closest to the region of maximum NIR laser absorption in the hydrogel, measure higher temperatures, while sensors which are further from the emitting tip measure lower temperatures.

The maximum temperature after 120 s of laser irradiation remains stable at around 14–15 °C, and after 1000 s of cooling the temperature returns to the initial value. Moreover, the spatial distribution of the temperature across the sensor length is not altered in the three cycles, and the heat transfer in time measured by each of the 10 sensors remains the same. These results are an indication of the good photothermal stability of the samples in these conditions and suggest the potential use of the Au NRs during sporadic irradiation for controlled delivery and cumulative release.^32,47^

In order to quantitatively evaluate the heating efficiency of the Au NRs in the given environment, the specific absorption rate (SAR) under 808 nm laser irradiation,^48^ which describes the energy converted into heat per time and mass, was calculated using eqn (1):1
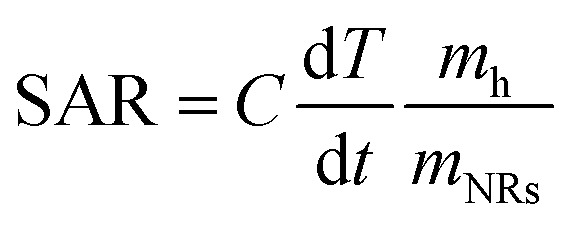
where *C* is the specific heat capacity of the hydrogel, considered equal to 4.18 J g^−1^ K^−1^; d*T*/d*t* is the initial slope of the time-dependent temperature curves shown in Fig. 5a; *m*_h_ is the mass of the hydrogel (calculated based on the measured density, equal to 1.01 g mL^−1^) and *m*_NRs_ is the mass of NRs suspended in the hydrogel.

For Au NR concentration equal to 0.00182 mg mL^−1^, the SAR is 1314 kW g^−1^, while for Au NR concentration equal to 0.00091 mg mL^−1^, the SAR is 1364 kW g^−1^, thus showing that SAR decreases when NR concentration increases. These results are in agreement with data regarding Au NPs provided in previous studies ^45,49^, indicating that the heating efficiency of Au NRs is the highest among many other NPs. The main differences can be ascribed to the values of the laser irradiance and Au NR concentration used in different studies.

## 4. Conclusions

The light source, in medicine, presents several advantages like minimal invasiveness, short treatment time and rapid recovery. In this framework, gold-based colloids showed great promise due to their high biocompatibility and responsive ability to NIR exposure. In the last few years, very good results were obtained with these nano-objects but several issues are still unsolved like the quick escape from the target site if they are not properly confined. The main consequences are the limit of the therapeutic effects on one side and accumulation problems in healthy tissues on the other. Hence, the aim of this study is to examine the NIR photothermal activity of gold nanorods confined within an agarose-carbomer (AC)-based hydrogel developed by our laboratory. The role of Au NRs once loaded within AC hydrogels was studied and discussed starting from highly photothermal efficient and stable Au NRs that have been designed and synthesized using a facile route. Based on the material analysis, we found that the Au NRs are stable also within hydrogel systems without being released for a long time. The NRs/HG device exhibited excellent thermal-enhanced NIR laser irradiation capability owing to the integration of the Au NRs within the three-dimensional network of AC hydrogels. These composite systems made of inorganic/organic combinations may facilitate the development of smart devices that can take advantage of the pros of both these systems.

## Conflicts of interest

There are no conflicts to declare.

## Supplementary Material

NA-005-D3NA00353A-s001
