# Supplementary material for: On the role of polymeric hydrogels in the thermal response of gold nanorods under NIR laser irradiation
Source: Nanoscale Adv. 2023 Aug 28;5(24):6870–9. doi: 10.1039/d3na00353a (PMC10696932; doi:10.1039/d3na00353a)
Supplement: NA-005-D3NA00353A-s001 [file NA-005-D3NA00353A-s001.pdf]

Electronic Supplementary Material (ESI) for Nanoscale Advances.
This journal is © The Royal Society of Chemistry 2023

Supporting Information

On the role of polymeric hydrogels in the thermal response of gold nanorods under NIR laser irradiation

Elisa Lacroce [a,1], Leonardo Bianchi [b,1], Laura Polito [c], Sanzhar Korganbayev [b], Alessandro Molinelli [a], Alessandro Sacchetti [a], Paola Saccomandi [b,2,*] and Filippo Rossi [a,2,*]

[a] Department of Chemistry, Materials and Chemical Engineering "Giulio Natta", Politecnico di Milano, via Mancinelli 7, 20131 Milan, Italy

[b] Department of Mechanical Engineering, Politecnico di Milano, via Giuseppe La Masa 1, 20156 Milan, Italy

[c] Consiglio Nazionale delle Ricerche, CNR-SCITEC, Via Giuseppe Fantoli 16/15, 20138 Milan, Italy

[1] equally contributed

[2] share the last authorship

* Paola Saccomandi, email: paola.saccomandi@polimi.it, Tel: +39-02-2399-8470

* Filippo Rossi, email: filippo.rossi@polimi.it, Tel: +39-02-2399-3145

Hydrogel characterization

Thermogravimetric analysis (TGA)

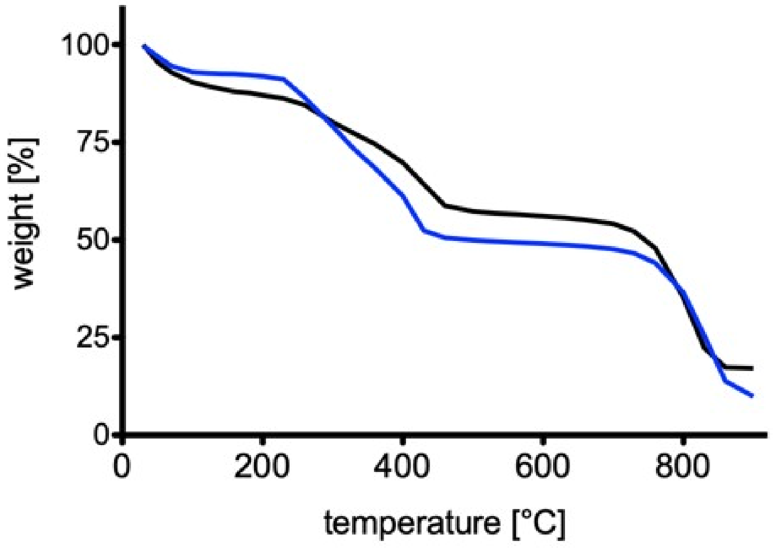

Figure S1. TGA analyses of Au NRs-loaded hydrogel samples (blue line) and control samples (black line).

Differential scanning calorimetry (DSC)

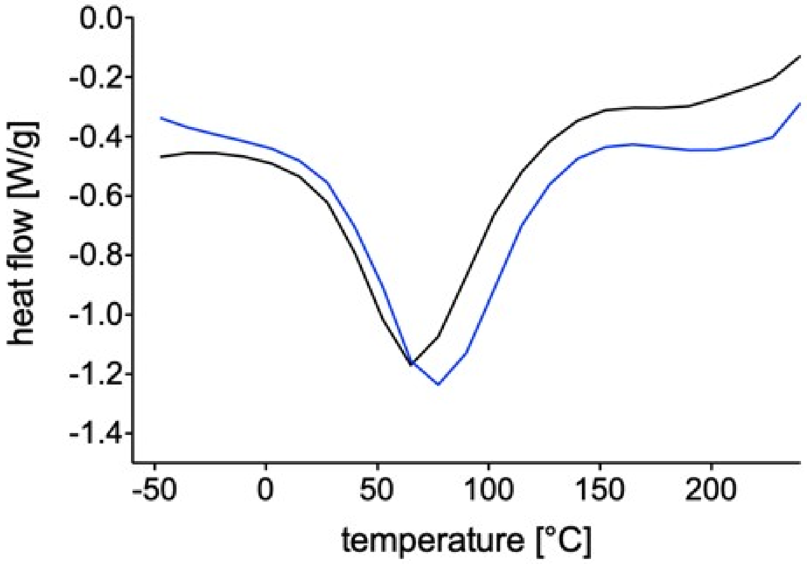

Figure S2. DSC analyses of Au NRs-loaded hydrogel samples (blue line) and control samples (black line).

Gelation time

	HGs	Au-HGs
gelation time [min]	7'10"	7'20"

Table S1. Gelation time of Au NRs-loaded hydrogel samples (Au-HGs) and control samples (HGs).

Scanning electron microscopy (SEM)

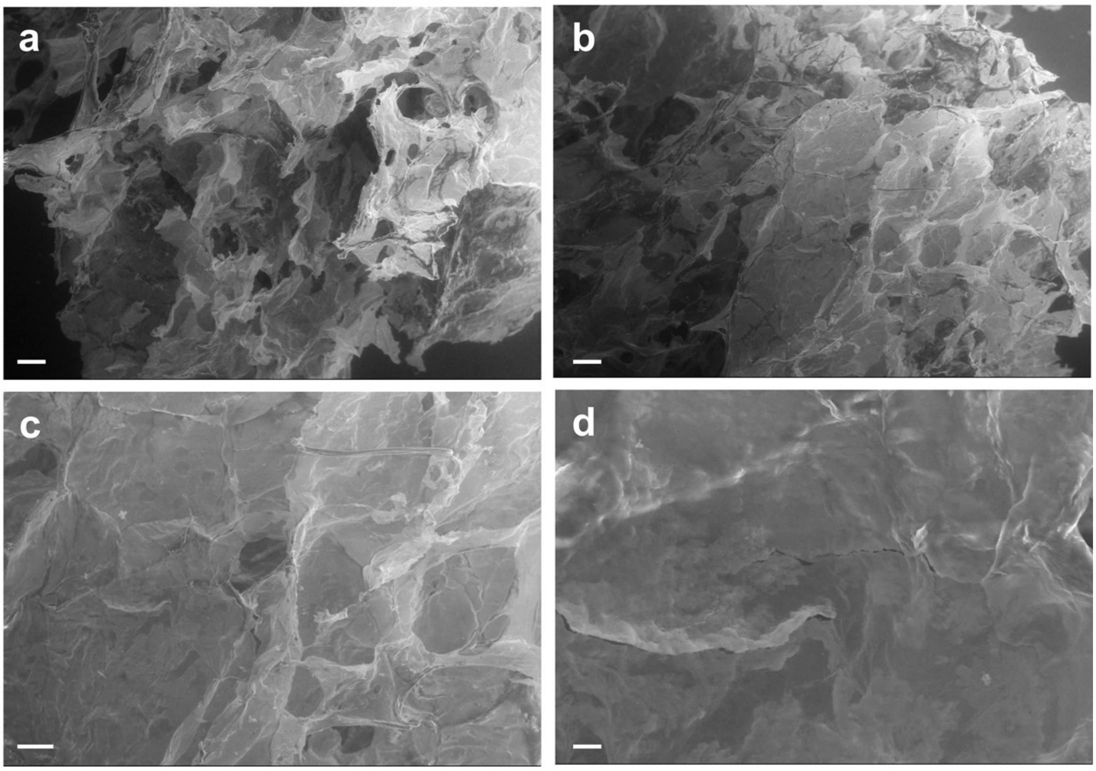

Figure S3. SEM analysis of AC hydrogels loaded with Au NRs (a, c) and neat AC hydrogels (b, d). Scale bars: 200 μm (a, b); 100 μm (c); 20 μm (d).
